# The Variability of the *Harlequin* Mouse Phenotype Resembles that of Human Mitochondrial-Complex I-Deficiency Syndromes

**DOI:** 10.1371/journal.pone.0003208

**Published:** 2008-09-15

**Authors:** Paule Bénit, Sergio Goncalves, Emmanuel Philippe Dassa, Jean-Jacques Brière, Pierre Rustin

**Affiliations:** Inserm, U676, Hôpital Robert Debré, Paris, and Université Paris 7, Faculté de médecine Denis Diderot, IFR02, Paris, France; Hospital Vall d′Hebron, Spain

## Abstract

**Background:**

Despite the considerable progress made in understanding the molecular bases of mitochondrial diseases, no effective treatments have been developed to date. Faithful animal models would be extremely helpful for designing such treatments. We showed previously that the *Harlequin* mouse phenotype was due to a specific mitochondrial complex I deficiency resulting from the loss of the Apoptosis Inducing Factor (Aif) protein.

**Methodology/Principal Findings:**

Here, we conducted a detailed evaluation of the *Harlequin* mouse phenotype, including the biochemical abnormalities in various tissues. We observed highly variable disease expression considering both severity and time course progression. In each tissue, abnormalities correlated with the residual amount of the respiratory chain complex I 20 kDa subunit, rather than with residual Aif protein. Antioxidant enzyme activities were normal except in skeletal muscle, where they were moderately elevated.

**Conclusions/Significance:**

Thus, the *Harlequin* mouse phenotype appears to result from mitochondrial respiratory chain complex I deficiency. Its features resemble those of human complex I deficiency syndromes. The *Harlequin* mouse holds promise as a model for developing treatments for complex I deficiency syndromes.

## Introduction

Most of the energy produced in the cell comes from oxidative phosphorylation (OXPHOS), which is catalyzed by the respiratory chain (RC) embedded in the mitochondrial inner membrane [1]. The respiratory chain comprises the proton-pumping respirasome [2] associating complexes I, III, and IV and a number of dehydrogenases including complex II. Electrons from respiratory substrates competitively converge from the various dehydrogenases to the CIII-associated quinone pool and are then conveyed to cytochrome oxidase and molecular oxygen by cytochrome *c* loosely bound to the mitochondrial inner membrane. The pumped protons are used to produce ATP from ADP and inorganic phosphate via the enzyme ATPase, which associates with adenylate and phosphate carriers to form the ATP synthasome [3]. OXPHOS dysfunction underlies a wide spectrum of human diseases, known as mitochondrial diseases. Respiratory chain complex I (RCCI) deficiency contributes 30% to 40% of all cases of mitochondrial disease [4]. Features of RCCI deficiency include optic atrophy, cerebellar ataxia, retinitis pigmentosa, growth retardation, and hypertrophic cardiomyopathy [5]. The mechanism, tissue-specificity, and course of mitochondrial diseases are poorly understood [6].

The number of reported human mitochondrial diseases has increased considerably in recent years. The underlying mutations affect either the mitochondrial DNA or the nuclear genes, of which more than 100 have been incriminated [7]. Treatments are extremely limited [8]. A major obstacle to the development of effective treatments is the absence of reliable animal models. Obtaining mice with mitochondrial DNA (mtDNA) mutations has proved challenging, and the animals rarely transmit the mutant mtDNA to their progeny, which limits their usefulness as tools for designing treatments [9]. Targeting nuclear genes encoding OXPHOS components has proved more efficient. However, extinction of a majority of the nuclear OXPHOS genes caused early embryonic death or produced no major phenotypic abnormalities. Cre-Lox recombination, in contrast, has been successful in producing mitochondrial abnormalities in specific tissue types. Thus, studies of Tfam-KO and frataxin-KO mice [10], [11] have shed light on a number of pathological mechanisms. However, the severe biochemical abnormalities in the target tissues fail to replicate human mitochondrial disease, limiting the value of these animal models for designing treatments. Recently, targeting the NDUFS4 subunit of complex I produced mice that exhibit several of the features seen in patients with RCCI [12]. However, the severe RCCI deficiency in these mice causes death at 7 weeks of age, and the animals do not exhibit the extraordinary variability in disease symptoms and course that characterizes human RCCI deficiency.

We recently established the occurrence of partial complex I deficiency in the Harlequin (*Hq*) mouse [13], which is characterized by progressive cerebellar ataxia [14]. Other features in these animals include early fur abnormalities, optic tract dysfunction with retinitis pigmentosa, and a risk of hypertrophic cardiomyopathy [14]–[16]. The *Hq* phenotype is due to a proviral insertion in the X-linked gene encoding the mitochondrial protein Apoptosis Inducing Factor (Aif) [14]. The insertion causes a partial decrease in the Aif protein to about 20% of the amount seen in wild-type mice. Gross development is not significantly affected, but the animals exhibit the typical features of mitochondrial disease. Total loss of Aif function caused abnormal cell death, presumably related to RCCI deficiency, during embryonic development but did not affect the temporal progression of patterning [17].

Although the involvement of Aif in RCCI assembly and stability is still being investigated [18], [19], the characteristics of the *Hq* mouse make it a potential model to investigate complex I deficiencies. With the aim to establish the value of this model, our main objective was to study in details the course of the abnormalities in *Hq* mice and the tissue-specific biochemical features at various time points. We found that the temporal progression of complex I deficiency differed across tissues and that the involvement of each tissue correlated with the residual amount of complex I 20 kDa subunit. Although oxidative injury has been suggested as a key mechanism in the *Hq* phenotype [16], antioxidant enzymes were normal except in skeletal muscle, where they were moderately increased.

## Results

### I. The *Harlequin* phenotype course

The onset of most of the gross phenotypic abnormalities varied widely across individuals. Although all the mutant animals eventually developed the disease phenotype, the time-course ranged from early ataxia and death to the slow development of functional impairment over several months. These differences were detected by monitoring body weight, fur abnormalities, and ataxia.

**Figure 1:**
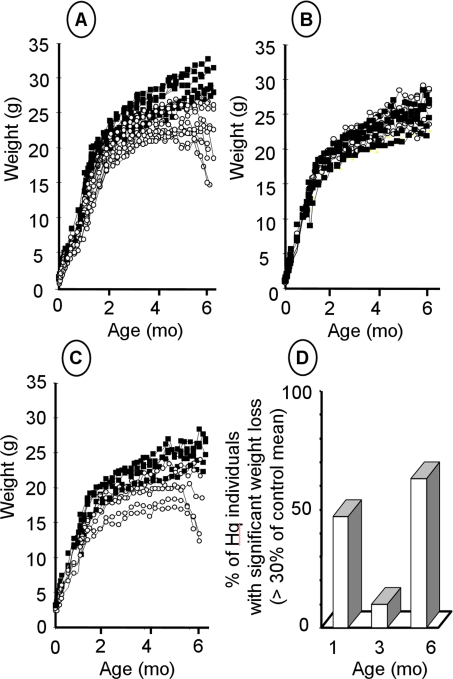
Time-course of weight loss in *Harlequin* mice. A: Weight changes with age in *Harlequin* hemizygous males (*Hq*/Y; n = 19) and wild-type males (B6CBACa Aw-J/A-Pdcd8 strain; n = 14). B, C: Weight changes with age in wild-type (n = 6) and heterozygous females (*Hq*/*Aif*+; n = 6) (B) and in homozygous females (*Hq*/*Hq*; n = 6) (C). D: Weight deficiency in *Hq* mice (n = 25) compared to wild type mice (n = 20) at various ages. Dark squares and open circles indicate control and *Hq* mice respectively.

Weight loss and growth retardation are common among patients with RC disorders [20], [21], we therefore first studied this parameter in wild type and *Hq* mice. B6CBACa Aw-J/A-Pdcd8 mice are relatively small. Thus, in our study, the wild type mice achieved their adult weight of 27±2 g for males (n = 19) and 22±2 g for females (n = 22) within 4 months. Body weight varied widely in the hemizygous *Hq*/Y males (Fig. 1A) and homozygous *Hq*/*Hq* females (Fig. 1C), with some *Hq* individuals having similar weights to those of wild type animals. No growth retardation occurred in the heterozygous females (Fig. 1B). At 1 month of age, about half the *Hq* males had marked growth retardation with a greater than 30% decrease in body weight compared to wild type animals (Fig. 1D). Most of these animals had normal body weights (within 30% of the control mean) at 3 months of age and significantly decreased body weights at 6 months of age compared to controls. However, 30% of *Hq* animals had no significant growth retardation at 6 mo of age. Growth retardation probably started before birth, as birth weight was significantly lower in the *Hq* population than in the wild type population (1.2±0.2 g (n = 15) and 1.5±0.2 g (n = 13) respectively; *p*<0.001). The sex ratio was normal in all the litters and the number of *Hq* individuals was consistent with Mendelian inheritance. About 30% of *Hq* animals died during the 6-month study, with most deaths occurring between 15 and 30 d of age. The only developmental abnormality found in the *Hq* population was ocular hypoplasia with absence of the optic nerve, which was noted in 40% of *Hq* individuals at 1 mo of age. Ocular hypoplasia occurred at the severe end of the disease spectrum and was present in many of the animals that died early on. At 3 and 6 mo of age, ocular hypoplasia was present in 20% and 10% of *Hq* animals, respectively. Ocular hypoplasia with absence of the optic nerve has been previously reported in human mitochondrial disease [22].

**Figure 2:**
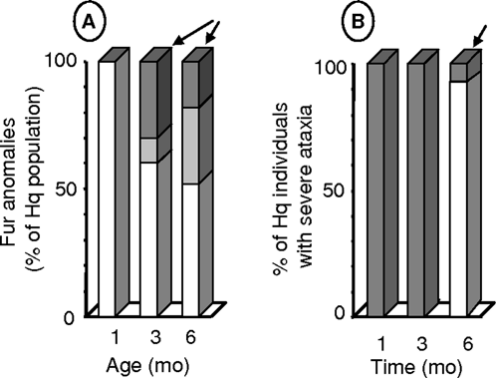
Variability of baldness and ataxia severity in *Harlequin* mice. A: Baldness in *Hq* mice. Open bars indicate severe baldness (more than 70% of the body surface area) at ages when controls had their full adult coat. Gray bars indicate patchy baldness (less than 30% of the body surface area). Patch distribution varied across animals. Dark bars represent animals with a full coat, many of which had sparser hair than the controls. The arrow shows that a substantial proportion of *Hq* mice had nearly normal hair at 3 and 6 mo of age. B: Severe ataxia in *Hq* animals (open bars; three or more falls in 5 minutes) was detected only at 6 mo of age. The arrow shows that two *Hq* animals had only mild ataxia at 6 mo of age.

Paucity of fur was first described as the hallmark of *Hq* mice [23]. We noted complete baldness in most of the *Hq* animals initially (Fig. 2A) followed by some hair growth resulting in a patchy or near-normal coat. At 3 mo of age, about half the *Hq* animals were bald over more than 30% of their body surface area and about 30% had near-normal fur. At 6 mo of age, 10% of *Hq* animals had near-normal fur.

Ataxia was detected in most of the animals at 3 mo of age. Severe ataxia defined as more than three falls within 5 minutes was noted only late in the disease, at 6 mo of age (Fig. 2B). At 3 mo of age a few animals had an unsteady gait but none had severe ataxia. At 6 mo of age, 90% of *Hq* males and females had marked gait ataxia but 8% had no clinical evidence of ataxia (Fig. 2B; arrow).

**Figure 3:**
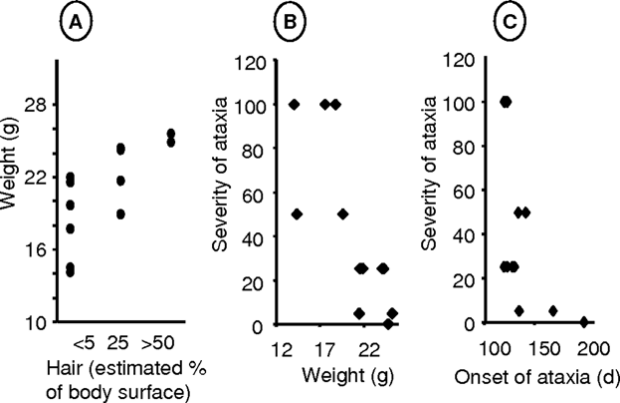
Correlations among the severities of the major abnormalities in *Harlequin* mice. A: Baldness (% body surface area) plotted against body weight in *Hq* males (n = 12). B, C: Ataxia (number of falls per min) plotted against body weight (B) or against the age at the first recorded sign of ataxia (C).

We looked for correlations linking growth retardation, baldness, and ataxia in the *Hq* population (Fig. 3, A–C). Plotting body weight against percentage of surface area without fur at 6 mo of age (Fig. 3A) showed that weight was highest in the animals that had nearly complete coats and vice versa. The animals with higher body weights were also those with less severe ataxia (Fig. 3B). Ataxia was more severe in the animals with earlier disease onset (Fig. 3C). Thus, body weight, baldness, ataxia, and age at onset seemed to vary in lockstep.

**Figure 4:**
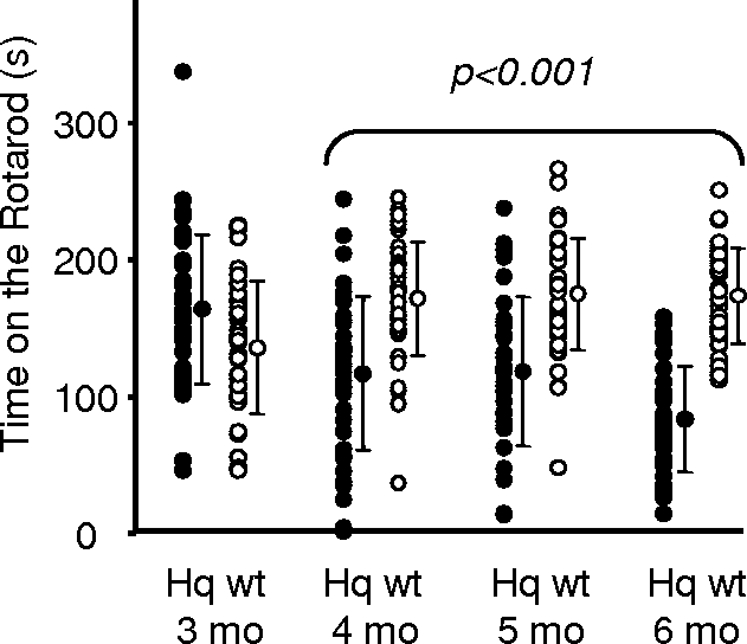
Variable Rotarod performance in control and *Harlequin* mice. Plots of individual results of three tests *per* animal (3 mo: 16 wild-type and 13 *Hq* mice; 4 mo: 11 wild-type and 13 *Hq* animals; and 5 and 6 mo: 15 wild-type and 11 *Hq* animals) showing the wide variability of values in both the *Hq* and control populations at each time point. The mean and standard deviation are shown for each distribution. Despite the major variability, *t*-tests showed significantly poorer performance in the *Hq* mice at 4 mo of age.

Motor coordination used as a rough estimate of neurological involvement was tested using a Rotarod device (Fig. 4). Performance varied widely in the *Hq* and wild type animals at 3, 4, 5, and 6 mo of age. A significant difference between *Hq* and wild type animals was noticeable from 4 mo of age onward, with some of the *Hq* animals performing very poorly at 4 mo of age.

### II. Complex I defect and its consequences in *Harlequin* mouse tissues

**Figure 5:**
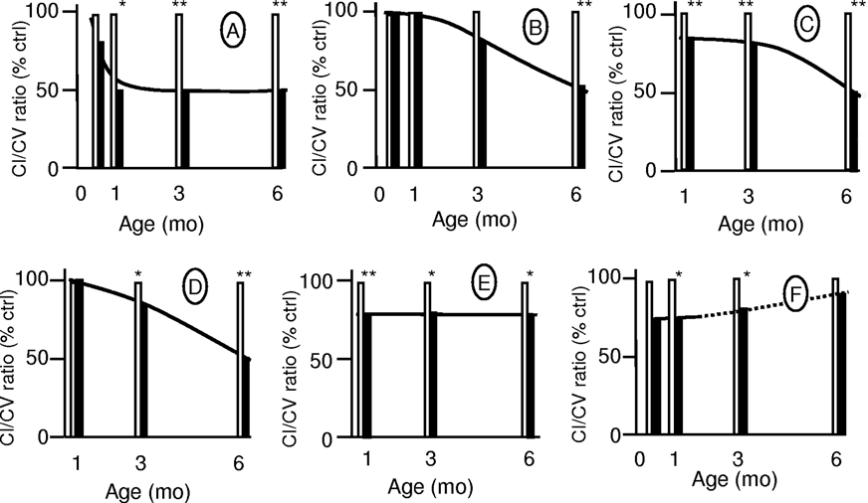
Differences in the time-course and severity of complex I deficiency across tissues from *Harlequin* mice. A–F: Complex I activity in homogenates prepared from cerebellum (A), thalamus (B), cortex (C), optic nerves (D), skeletal muscle (E), and retinas (F). Complex I activity was measured by spectrophotometry [40] and normalized for the activity of complex V, which was not affected by Aif depletion. The open and dark symbols refer to the control and *Hq* mice, respectively. Continuous lines indicate changes in complex I activity over time in various tissues of *Hq* mice. The dotted line indicates changes in complex I activity over time in *Hq* retinas, presumably ascribed to biased sample collection. Numbers of animals were as follows: 15 d of age, 3 controls and 3 *Hq* mice; 1 mo of age, 5 controls and 5 *Hq* mice; 3 mo of age, 5 controls and 5 *Hq* mice; 6 mo of age, 10 controls and 10 *Hq* mice. *p<0.005; **p<0.001.

We previously reported partial loss of complex I activity by about 40% of the value in controls in the whole brain of *Hq* mice, with variations in the severity of the deficiency across tissues and cells [13]. Here, we show that complex I activity was about 50% of the control value in the cerebellum of *Hq* mice (Fig. 5A). At 6 mo of age, complex I deficiency was noted in all the brain territories investigated (cerebellum, thalamus, and cortical-enriched fraction), as well as the optic nerves (50% residual activity) and retinas (25% residual activity) (Fig. 5A–D). Complex I deficiency was detected at 1 mo of age, before the occurrence of cerebellar atrophy or major ataxia (Fig. 5), in any of the 20 *Hq* animals at this time point. In other organs, complex I activity was normal (heart, liver, and testis) or only slightly reduced (by 20% in the spinal cord and 10% in the kidney). Complex I activity was decreased by about 30% in the skeletal muscle (Fig. 5E). In most of the affected tissues, complex I deficiency worsened over time, albeit with a variable course (Fig. 5). The deficiency reached its greatest level by 1 mo of age in the cerebellum and skeletal muscle. In the thalamus, cortex, and optic nerve, in contrast, the deficiency was modest or absent at 1 mo of age and reached 50% by 6 mo of age. Complex I activity in the retinas was decreased by about 30% by 1 mo of age but improved subsequently, probably as a result of bias due to gradual selection of animals with normal eyes.

**Figure 6:**
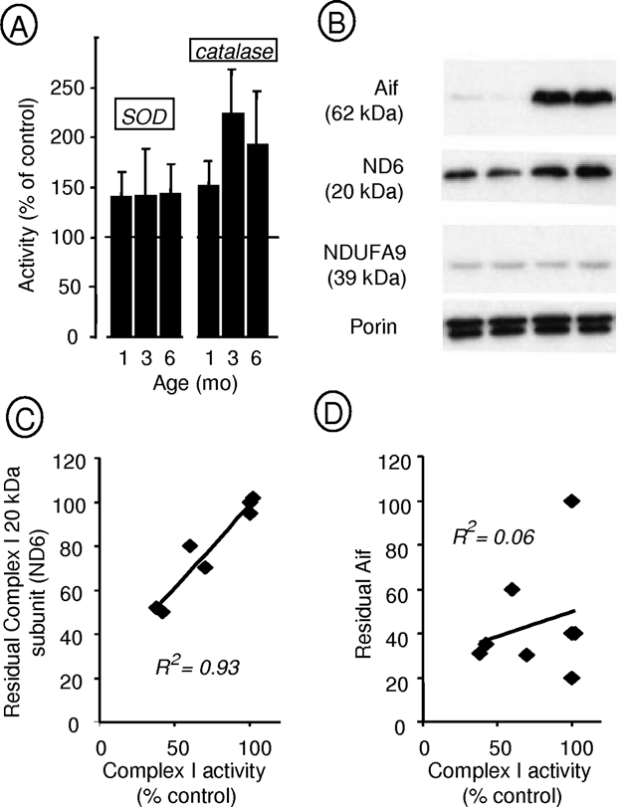
Antioxidant enzymes and Complex I 20 kDa subunit in various tissues of *Harlequin* mice. A: Superoxide dismutase (manganese- and copper, zinc-dependent SODs) activity and catalase activity measured as described in Materials and Methods in skeletal muscle tissue from *Hq* mice at various ages. B: Western blot analysis of Aif, complex I 20 and 39 kDa subunits (ND6 and NDUFA9, respectively) and porin in skeletal muscle of *Hq* mice (lines 1, 2) and control mice (3, 4). C: Plot of residual complex I 20 kDa subunit in mouse tissues against residual complex I activity, showing a significant correlation. D: Plot of residual Aif in mouse tissues against residual complex I activity, indicating no significant correlation.

Oxidative insult has been suggested as a key factor in the pathogenesis of the *Hq* phenotype [16]. Superoxide dismutase (SOD) and catalase activities are induced by oxidative insult *in vivo*. We compared SOD and catalase activities at 6 mo of age in control (n = 10) and *Hq* (n = 10) mice (Fig. 6A). Moderate delayed increases were found in skeletal muscle (to about 150% and 200% of control values for SOD and catalase, respectively, at 6 mo of age). However, the activities of these two enzymes were not increased in any of the other tissues investigated in this study.

Since the extent of complex I deficiency varied across tissues, we measured residual complex I using the 20 kDa subunit of complex I as a marker. We reported previously that this subunit, now recognized as NDUFB8 (see Material and Methods), was decreased in case of Aif deficiency [24]. Western blot analysis of skeletal muscle samples from *Hq* mice showed a 60% decrease (Fig. 6B) in the 20 kDa subunit compared to controls, indicating a 30% decrease in complex I activity (Fig. 5E). Western blot analysis was also performed with samples of heart, liver, kidney, cerebellum, cortex, and spinal cord. We plotted the residual complex I activities in these tissues, as measured previously, against residual 20 kDa subunit (Fig. 6C). A significant correlation was found between these two parameters. In particular, tissues without complex I deficiency (*e.g.* heart, liver) contained normal amounts of 20 kDa subunit.

**Figure 7:**
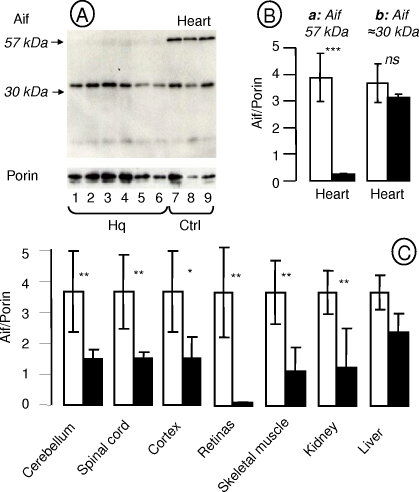
Aif in Harlequin mice. A: Western blot analysis of Aif in the heart of *Hq* mice (lines 1–6) and control mice (lines 7–9). In the heart, two bands reacted with the Aif antibody. The band with the highest molecular weight was produced by the Aif1 isoform (molecular weights of the unprocessed and processed forms, 67 and 57 kDa, respectively). The molecular weight of the other band is consistent with the Aif sh2 isoform. This second form was not detected in any of the other tissues studied (cerebellum, spinal cord, cortex, retinas, skeletal muscle, kidney, or liver). Porin was used as a loading marker. B: Proportion of the two Aif isoforms identified in the heart of control mice (open symbol) and *Hq* mice (dark symbol) mice. C: Aif (57 kDa) content in various tissues of control and Hq mice normalized for porin content. **p<0.005; ***p<0.001.

We measured Aif content in various tissues of *Hq* mice and controls (Fig. 7). In all studied tissues, one major (>98%) band at 57 kDa reacted with antibody to the Aif internal region. Heart samples from control animals showed two bands, at 57 and about 30 kDa, in nearly identical proportions (Fig. 7A). In heart samples from *Hq* animals, in contrast, the 57 kDa was extremely faint (Fig. 7A–B). When we compared the residual amount of Aif (57 kDa) in various tissues of *Hq* mice (n = 6) and controls (n = 3), we found that Aif was barely detectable in the retinas and was reduced to 20%–40% in most tissues except for the liver, where the level was about 60% of the control value. We found no correlation between the amount of residual Aif protein and tissue involvement (Fig. 6D).

## Discussion

The *Hq* phenotype is caused by severe Aif protein deficiency in hemizygous males and homozygous females. Aif gene impairment is due to an ecotropic proviral insertion in the first intron of the gene, as shown by Klein et al. [14]. Aif deficiency leads to RCCI deficiency in affected tissues of *Hq* mice [13], in ES cells [13] and embryos [17] of Aif knock-out mice, in Aif knockdown human HeLa cells [13], in mouse tissues (skeletal muscle, heart, liver) with tissue-specific ablation of the Aif gene [24], [25], and in Aif knockout *Drosophila* [26]. These conditions are characterized by RCCI deficiency without evidence of oxidative stress except in the skeletal muscle, where SOD and catalase activities are increased [24]. Evidence of antioxidant enzyme induction has led to the suggestion that Aif may protect the mitochondria against oxidation [16]. Primary complex I deficiency has been reported to result in oxidative stress [27], but there is no evidence that oxidative stress can specifically cause complex I deficiency. Impairment of mitochondrial antioxidant enzymes (*e.g.*, manganese-dependent SOD) results instead in a generalized deficiency of the RC, including the iron-sulfur cluster-containing enzymes (complexes I, II, and III) and the Krebs cycle enzyme aconitase [28]. Accordingly, treatment of Aif-depleted human cells (Hep3B and HeLa cell lines) with antioxidants (*N*-acetyl-cysteine or MitoQ) failed to restore complex I integrity [29], suggesting primary loss of complex I, which might increase superoxide leakage. Conversely, the (pro-oxidant) superoxide-generating NADH oxidase activity of Aif may be required for normal muscle regeneration [30]. Taken in concert, the data suggest that any antioxidant effects of Aif may be confined to the close vicinity of complex I, so that Aif acts as a complex I maintenance protein. Antioxidant enzyme induction in muscle-specific Aif knockout mutants [24] and in skeletal muscle from our *Hq* mice appears to be tissue-specific and may reflect either an additional role for Aif in muscle or a tissue-specific consequence of RCCI. Although the role for Aif remains debated [26], [29], [30], the effects of Aif deficiency are similar to those of deficiency in any complex I assembly/maintenance factor. NDUFS4 knockout mice exhibit severe RCCI with features similar to those of *Hq* mice (growth retardation, blindness, ataxia, and baldness) but greater disease severity leading to death at 7 weeks of age after only 2 weeks with symptoms. Thus, complex I deficiency seems to be the key determinant of the *Hq* and NDUFS4 knockout models.

Multivisceral disease develops in *Hq* mice over time. As with human complex I-associated diseases, the brain and optic tract are affected [5], whereas the liver is spared. We failed so far to obtain a clear analogy between the standard Leigh syndrome images observed in a number of patients with complex I deficiency and the *Hq* mice. Similarly, T2-weighted MRI images showing signal hyper intensity have not been reported for the NDUFS4 mutant mice. Disease progression follows the same pattern in all NDUFS4 knockout mice but varies considerably across *Hq* mice. As with humans, both genetic and non-genetic factors may explain this variability, since the animals do not have a pure genetic background (B6CBACa Aw-J/A-Pdcd8) and the phenotypic consequences of mitochondrial dysfunction depend on a number of unidentified factors. Marked inter-individual variability also occurs in human mitochondrial diseases. Thus, the *Hq* model, although difficult to study, constitutes a good model of human mitochondrial diseases. Obviously, as long as the intimate mechanism linking Aif depletion and impaired complex I deficiency is not fully depicted, this model will be best used to test for efficiency of compounds/strategies aiming at counterbalancing the consequences of complex I deficiency.

Reduced birth weight was the earliest abnormality in *Hq* animals compared to their wild type littermates and suggested intra-uterine growth retardation (IUGR). Similarly, *Aif*-KO mouse embryos had severe growth retardation contrasting with a normal temporal progression of patterning [31]. Among humans with RCCI deficiency, 23% had low birth weights [20]. In mice, complete Aif gene deletion not only caused early deaths by embryonic day E9 related to RCCI deficiency [32], but also resulted in marked embryonic growth retardation [17]. Body weight in our *Hq* animals was low at birth and subsequently remained lower than in the control animals. The mechanism of IUGR associated with RC deficiencies remains unclear. In humans, early antenatal expression of respiratory chain deficiencies (including RCCI deficiency) reflects the time-course of disease-causing-gene expression *in utero* [20]. *In utero*, respiratory chain deficiency may result in decreased ATP formation and/or in alteration of apoptotic events controlled by the mitochondria.

Hair abnormalities occur in a substantial proportion of human patients with mitochondrial disorders. In 8 of 25 children with mitochondrial diseases, slow-growing, sparse, fragile hair was an early manifestation [33]. This fact, together with the hair abnormalities seen in NDUFS4 knockout mice, supports a role for RCCI deficiency in the hair abnormalities of *Hq* mice. The correlation in our *Hq* mice between the severity of baldness and the severity of growth retardation also suggests a role for RCCI deficiency in the hair abnormalities, as delayed growth is probably due to RCCI deficiency.

The severity of cerebellar ataxia varied widely in our *Hq* mice, probably reflecting a variable degree of neuron loss. Similar variability occurs in humans with mitochondrial disorders [6]. The greater severity of ataxia at 6 mo of age in a subset of our *Hq* animals compared to an earlier study [14] suggests a role for unidentified environmental factors and a possible influence of the mixed genetic background. Interestingly, complex I deficiency in the cerebellum was readily evidenced at 15 d of age, whereas cerebellar ataxia was detected only after several weeks in the most severely affected animals. Thus, cerebellar function persisted for several weeks despite the presence of complex I-deficient mitochondria.

Our study does not shed new light on the mechanism of the tissue specificity associated with complex I deficiency. However, our data establish that residual complex I measured using the 20-kDa subunit as a marker correlates with the residual complex I activity in affected tissues. In contrast, residual complex I activity was not correlated with residual Aif protein. Five Aif isoforms have been identified to date [34], suggesting a possible explanation for the variability of tissue involvement. In control hearts, a shorter isoform contributes as much as 50% of total Aif. The normal complex I activity in the heart may be ascribable to the presence of this short Aif isoform, whose electrophoretic properties resemble those of the Aif-sh 2 [34].

Taken together, our results indicate that *Hq* mice replicate the main features of RCCI deficiency in humans, including tissue-specificity, course, and interindividual variability. The *Hq* model is likely to prove valuable for investigating treatments aimed at RCCI deficiency.

## Materials and Methods

### Animals

Homozygous (*Hq*/*Hq*) females and hemizygous (*Hq*/Y) males were obtained by mating *Hq*/X females with either *Hq*/Y or control males obtained from The Jackson Laboratory (Bar Harbor, ME). The *Hq* strain was B6CBACaA^w-J^/A-Pdc8*^Hq^*/J. All mice used in this study were F1 mice bred from founders having a mixed genetic background. The mice were housed with a 12-h light/dark cycle and free access to food (3% lipids, 16% protein; SAFE A-04 chow; UAR Epinay sur Orge, France) and water.

### Genotype determination

Mice were genotyped using multiplex PCR with five primers: two for sex determination (SRY: 5′-TGGGACTGGTGACAATTGTC-3′ and 5′-GAGTACAGGTGTGCAGCTCT-3′), two for the wild-type *Aif* allele (*Aif* 1F: 5′AGTGTCCAGTCAAAGTACCGG-3′; *Aif* 1R: 5′-CTATGCCCTTCTCCATGTAGTT-3′), and one for the *Aif* allele harboring the proviral insertion (*Hq* allele) (*Aif* RV: 5′-CCCGTGTATCCAATAAAGCCTT-3′).

### Phenotype determination

Three hallmarks of the *Hq* phenotype [14] were studied: baldness, growth retardation, and cerebellar ataxia. Baldness was assessed as the percentage of body surface area without hair. Weight was determined daily from birth through day 7 of postnatal life and weekly thereafter. Cerebellar ataxia was assessed as the number of falls per minute of walking, with severe ataxia being defined as three or more falls within 5 minutes; and by testing on a computer-driven Rotarod device (Imetronic; Pessac, France). The mice were trained at 3, 4, 5, and 6 mo of age, for 3 consecutive days after a previous day of training, at speeds that increased from 4 to 40 rpm/min.

### Tissue homogenate preparation and enzyme assays

Frozen tissue (cortex, cerebellum, thalamus, spinal cord, optic nerve, retinas, heart, liver, kidney, skeletal muscle, testis) homogenates were prepared using a 1 ml glass-glass potter in 500 µl of extraction buffer composed of 0.25 mM sucrose, 40 mM KCl, 2 mM EGTA, 1 mg/ml BSA, and 20 mM Tris-HCl (pH 7.2). Large cellular debris was separated by centrifugation (1500 *g*×5 min). RCCI activity was measured as recently described using a Cary 50 spectrophotometer (Varian Australia, Victoria, Australia) [35]. Catalase activity and total SOD level were quantified as already described [36], [37]. Protein was estimated using the Bradford assay [38].

### Western blot analysis

Western blot analysis was performed on mitochondria-enriched supernatant (900 g×5 min) prepared from tissue homogenate separated by SDS-PAGE, and blotted onto a PVDF membrane. The membranes were blocked for 1 h in TBST tween 0.01% and then incubated for 1–3 h with different antibodies (AIF1∶1000, chemicon; complex I subunits 20 kDa and 39 kDa∶1∶1000, Molecular Probes and porin 1∶1000, MitoSciences. It must be mentioned that future controversy about the identity of the 20 kDa antibody may occur, because different lines of evidence suggest that, in fact, the protein recognized by the antibody provided by Molecular Probes is not ND6, but a different complex I membrane arm subunit, NDUFB8, with a similar molecular weight as ND6 (Dr Jose Antonio Enriquez, University of Zaragoza, Spain, personal communication to Dr Fernandez-Moreira) [39]. The horseradish peroxidase-conjugated secondary antibody (1∶5000) was incubated for 1 h at room temperature in 5% milk powder in TBST buffer, 0.01% Tween 20. Peroxidase activity was visualized with the ECL plus (GE healcare) according to the manufacturer's instructions. Chemiluminescent signal were captured on autoradiography and used to assess protein content. The relative protein levels of AIF and Complex I 20 kDa subunit were assessed by their chemiluminescent signals compared with that of the porin protein.

### Statistics

We evaluated mean differences in study variables between *Hq* and wild-type populations by using appropriate t-tests (SigmaStat software, Richmond, CA) to compute the means, standard deviations, and *p* values as indicated. Values of *p* less than 0.001 were considered statistically significant.
